# Variable number tandem repeats – Their emerging role in sickness and health

**DOI:** 10.1177/15353702211003511

**Published:** 2021-04-01

**Authors:** Jack NG Marshall, Ana Illera Lopez, Abigail L Pfaff, Sulev Koks, John P Quinn, Vivien J Bubb

**Affiliations:** 1 Department of Pharmacology and Therapeutics, Institute of Systems, Molecular and Integrative Biology, University of Liverpool, Liverpool L69 3BX, UK; 2 Perron Institute for Neurological and Translational Science, Perth, WA 6009, Australia; 3 Centre for Molecular Medicine and Innovative Therapeutics, Murdoch University, Perth, WA 6150, Australia

**Keywords:** VNTR, SVA, transcriptional regulation, neurological disease, physiology

## Abstract

Understanding the mechanisms regulating tissue specific and stimulus inducible regulation is at the heart of understanding human biology and how this translates to wellbeing, the ageing process, and disease progression. Polymorphic DNA variation is superimposed as an extra layer of complexity in such processes which underpin our individuality and are the focus of personalized medicine. This review focuses on the role and action of repetitive DNA, specifically variable number tandem repeats and SINE-VNTR-*Alu* domains, highlighting their role in modification of gene structure and gene expression in addition to their polymorphic nature being a genetic modifier of disease risk and progression. Although the literature focuses on their role in disease, it illustrates their potential to be major contributors to normal physiological function. To date, these elements have been under-reported in genomic analysis due to the difficulties in their characterization with short read DNA sequencing methods. However, recent advances in long read sequencing methods should resolve these problems allowing for a greater understanding of their contribution to a host of genomic and functional mechanisms underlying physiology and disease.

## Impact statement

Interpretation of the functional consequences of human genome variation is an essential part of modern biomedical research. This review describes the impact of repetitive DNA on experimental biology by allowing insight into the role of non-coding DNA in regulation of gene structure and function. The genetic variation inherent in repetitive DNA is becoming an increasing important parameter in personalized medicine. Emerging long read DNA sequencing technologies should aid the improved characterization of these elements in the human genome which will integrate that knowledge into clinical practice and improve the precision of clinical diagnoses and decision-making. This review should prove a valuable resource to the field to capture the distinct mechanism utilized by repetitive DNA in gene function.

## Introduction

Ninety-eight percent of the human genome does not code for proteins, but contains a variety of regulatory elements including those to direct (a) tissue specific and stimulus inducible gene expression, (b) differential mRNA splicing to generate distinct protein profiles or turnover, and (c) functional non-coding RNAs. The properties of these regulatory components like the exons themselves can be modified by genetic polymorphism or mutations. To date, genome analyses have favored short read next generation DNA sequencing and genome-wide association studies (GWAS). The latter has focused on analyzing variation in single nucleotide polymorphisms (SNPs) and has identified genomic variation in strong association with phenotype both in exons and non-coding regions. Such SNPs in exons are often easy to interpret mechanistically as many will change a key amino acid providing an explanation for the phenotypic association. However, the functional significance of the vast majority of SNPs in non-coding DNA is difficult to determine; furthermore, a major shortfall of this approach is that it has neglected the role and function of other sources of genetic variation in the genome such as that represented by repetitive DNA. This is in part due to the inherent instability of human repetitive DNA in *E. coli* cloning strategies used in the construction of the library of DNA sequences incorporated into the reference genome, and the difficulties in interpreting and reassembling sequence from such short read sequences in areas of repetitive DNA. The advent of long-read sequencing technologies will help overcome the latter problem, and technologies such as Oxford Nanopore or Pacific Biosciences single-molecule real-time sequencing (PacBio) can easily read 10 kb or more sequence; furthermore, base calling accuracy has improved (error rate said to be <1% for PacBio and <5% for Oxford Nanopore) and analytical pipelines to call structural variation have been developed.^1^ This review will focus on one class of repetitive DNA, namely variable number tandem repeats (VNTRs), highlighting their functional roles in genome regulation and as biomarkers of disease focusing on neurological diseases and disorders

## Consequences and genetic nature of tandem repeat DNA

Tandem repeat (TR) DNA imparts a huge source of variation to the genome, and TRs have evolved throughout evolution and contribute to genetic diversity. Although they are found in many eukaryote species, present day interest has focused on their role in hominid evolution and their contribution to the development of human-specific traits in the evolution of modern humans and in particular neural function.^2–4^ In modern humans, TR DNA has been associated both with traits such as aggression and addictive behaviors, reviewed by literature^5–8^ and implicated in the increased susceptibility to, and risk of developing, a wide variety of neurological diseases such as motor neuron disease^9–11^ and Alzheimer’s disease ^12,13^ mental health conditions such as bipolar disorder, depression, and schizophrenia.^14,15^ To date, 1584 such repeats have been found to be human specific and it has been proposed that they contribute to human-specific traits.^4^ When the repeat number is variable, i.e. polymorphic, they become VNTRs, a class which accounts for approximately 3% of the human genome^16,17^; such repeat elements can vary in copy number and additionally demonstrate single nucleotide variation within the repeats or even short insertions or deletions. There are several mechanisms proposed, by which this variation could develop, including errors in DNA replication such as slipped-strand mispairing which results in the misalignment of DNA strands and thus expansion or contraction of the copy number of the DNA motifs,^18^ homologous recombination, and duplications. Recently it has been shown 55% of VNTRs map to the terminal 5Mbp of human chromosomes,^19,20^ and regions that have previously been found to demonstrate increased double-strand breaks during early stages of meiosis^21,22^ and male meiotic recombination. VNTRs may be located within exons, introns, or intergenic spaces and this aforementioned group of VNTRs appears to favor intronic locations.^4^ A second pathway contributing to the derivation of VNTRs is via retrotransposition, particularly utilizing the composite SINE-VNTR-*Alu* (SVA) element, and these elements tend to show bias against incorporation in to genic regions, and have a high GC content.^4^ Unsurprisingly, VNTRs located within exons are the smallest group. Originally the main focus of research was on short tandem repeats (STR) (or micro-satellites) as it became apparent that expansions of these can occur in somatic cells, and this phenomenon is implicated in diseases such as fragile X-associated tremor/ataxia syndrome, myotonic dystrophy, Huntington’s disease, spinocerebellar ataxia, and amyotrophic lateral sclerosis (ALS).^23–33^ This approach originally identified a number of diseases associated with triplet expansions, i.e. Fragile X syndrome in 1991 ^23^ and Huntington’s disease in 1993;^34^ however, expansions of longer repeat sequences have since been found. For the latter, examples include: pentanucleotide expansions in the genes *SAMD12* and *RFC1* found in benign adult familial myoclonal epilepsy type1 and ataxia syndromes, respectively,^35,36^ a hexanucleotide repeat expansion in the *C9ORF72* gene associated with ALS,^32,33^ and a 12-nucleotide expansion in the *CSTB* gene associated with progressive myoclonic epilepsy of the Unverricht-Lundberg type or EPM1 ^37^ (Table 1). To date, incidences of personal somatic expansions or generation-to-generation instability in TRs of much larger repeat length have not been identified other than the intriguing recent report of an expanded VNTR sequence (around 25 nucleotides in length) in an intron of the *ABCA7* gene, and the repeat number ranges from 12 to 427 or greater, and has been identified as a risk factor in Alzheimer’s disease; however, it has not been reported whether the repeat number seen in this expansion is stable between germ and somatic cells from the same individual.^13^ In general, based on current sequence information, the polymorphic but stable expansions seen in longer TRs appear to pre-date the evolution of modern human populations and have been used to track ancient population migrations. Furthermore, for some VNTRs, the allele frequencies or base pair composition of the repeat unit have been found to differ between populations and ethnic groups throughout the world including *TRIB3,*^47^ *WRD7*,^42^ and *DNAJC5/miR-941.*^48^ This is a factor which must be taken into account when assigning “risk” to such alleles as exemplified by the hexanucleotide expansion associated with ALS in the *C9ORF72* gene which is one of the four most common causes of ALS in Caucasian populations but is much rarer in Chinese and other East Asian populations.^39,49^

**Table 1.** Examples from the text of neurological diseases and disorders which have been associated with polymorphic tandem repeats.

Gene	STR/VNTR/SVA	Disease association	Refs
*FMR1*	STR (triplet)	Fragile X-associated tremor/ataxia syndrome	^23,24^
*DMPK*	STR (triplet)	Myotonic dystrophy type 1	^28,29^
*HTT*	STR (triplet)	Huntington’s disease	^30,34^
*ATXN1*	STR (triplet)	Spinocerebellar ataxia type 1	^11,31^
		amyotrophic lateral sclerosis	
*NIPA1*	STR (triplet)	Hereditary spastic paraplegia type 6, amyotrophic lateral sclerosis	^9^
*NOTCH2NLC*	STR (triplet)	Neuronal intranuclear inclusion disease-related disorders, Parkinson’s disease	^38^
*SAMD12*	VNTR(pentamer)	Benign adult familial myoclonal epilepsy type1	^35^
*RFC1*	VNTR (pentamer)	Ataxia syndrome	^36^
*C9ORF72*	VNTR (hexamer)	Amyotrophic lateral sclerosis	^10,32,33,39^
		frontotemporal dementia	
*CSTB*	VNTR (12 mer)	Progressive myoclonic epilepsy of the Unverricht-Lundberg type or EPM1	^37^
*ABCA7*	VNTR (approx.25 mer)	Alzheimer’s disease	^13^
*SLC6A3*	VNTR (40 mer)	Attention-deficit hyperactivity disorder	^40,41^
*DRD4*	VNTR (48 mer)	Attention deficit hyperactivity disorder, addictive, and eating disorders	^5,8^
*WRD7*	VNTR (69 mer)	Amyotrophic lateral sclerosis	^42^
*SLC6A4*	VNTR (22–23 mer and 16–17 mer)	Anxiety-related traits	^7,14,43^
		Affective disorders	
*MAOA*	VNTR (30 mer and decamer)	Impulsive/anti-social/aggressive behaviors, affective disorders	^6,44,45^
*TAF1*	SVA	X-linked dystonia Parkinsonism	^46^

## Assessing the functional potential of VNTRs as transcriptional regulators of gene expression

An increasing number of VNTRs have been identified which support differential gene expression both *in vivo and in vitro.* Perhaps one of the earliest and most striking illustrations of allele-specific activity, was the *in vivo* demonstration of the ability of the human-specific *SLC6A4* intron 2 VNTR to direct differential reporter gene expression in the midbrain of mouse embryos equivalent to the area where the mouse serotonin transporter is initially expressed in the developing brain.^43,50^ Association studies have established a link between repeat copy number in this VNTR as a risk factor in various neurological diseases or disorders; however, establishing the functional significance of such VNTR polymorphism *in situ* is more problematic. Some of this difficulty may be attributed to non-coding VNTRs being regulatory domains that are only functional in specific tissues, developmental stages, or in response to specific cellular challenges. Recent examples of VNTRs found to have regulatory properties include the finding that the longer risk alleles of the intronic VNTR in *ABCA7* favored use of a cryptic splice site resulting in exon skipping ^12^ and that the number of VNTR repeats in the promoter region of *TRIB3* correlated with mRNA levels in various tissues.^47^

If VNTRs are involved in transcriptional or post transcriptional gene regulation then they will act in consort with other gene regulatory domains to direct tissue specific and stimulus inducible gene expression. For example, it has been shown that a VNTR located at the *MIR137* locus works together with the SNP rs2660304 to drive differential promoter activity *in vitro*, thereafter it was shown using haplotype analysis at the *MIR137* locus that rs2660304 was a proxy SNP for the schizophrenia GWAS SNP rs1625579.^51^ Furthermore, interactions between multiple VNTRs to regulate gene expression are possible. *In vitro* analysis has demonstrated that distinct VNTRs within a gene locus can act either independently or synergistically to regulate transcription. For example, the serotonin transporter (*SLC6A4*) gene which has VNTRs located both in the linked polymorphic region of the 5ʹ promoter and intron 2 which have been shown to act combinatorically,^52^ a further example is the *MAOA* gene which in addition to the well-characterized µVNTR has a second distal (d)VNTR^44^ located approximately 500 bp upstream of the µVNTR.^53^ The complexity this produces was illustrated by the recent meta-analysis of *MAOA* by Tunbridge *et al*. where it was found that the high and low activity µ alleles were associated with enzyme activity in the blood but did not affect MAOA mRNA abundance;^45^ concurrently, Manca *et al*. demonstrated that the distal VNTR regulated mRNA levels of the canonical isoform of MAOA in a cell line model.^54^ Moreover, specific regulatory effects on MAOA isoforms and an additive effect of the two VNTRs at this locus were highlighted in additional work.^53^ A further layer of intricacy was uncovered by the finding that VNTR (allele) specific responses to a stimulus also existed.^54^ Thus, it is important to consider the overall haplotype when analyzing gene regulation rather than assuming a single regulatory variation is solely associated with a specific phenotype.^47,53^

## Proposed mechanisms of action and emerging novel roles for VNTRs to alter the genomic transcriptome

**Figure 1.**
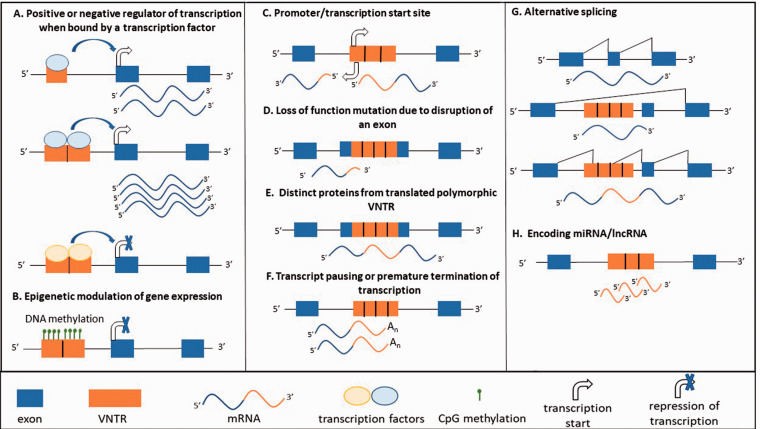
Mechanisms by which tandem repeat DNA can modify gene expression. Differential regulation at an allele can result either as a result of polymorphic repeat number, SNPs or indels in the repeats, pathogenic expansion of the repeat or in the case of SVAs presence or absence polymorphism. (A color version of this figure is available in the online journal.)

In addition to the more conventional mechanism of provision of transcription factor (TF) binding domains by which VNTRs can contribute to the regulation of transcription, there are several examples of them encoding miRNAs and affecting epigenetic parameters (Figure 1). In general, VNTRs have been shown to function as transcriptional regulatory domains, by providing binding sites for transcription factors and modulating the affinity of binding on the basis of repeat unit copy number.^55–57^ More specifically in the context of neurological disorders, the previously mentioned *SLC6A4* intron 2 VNTR has been assessed via reporter gene assays in rat prefrontal cortical cells, where it was found that the different copy number variants induced reduced but differential reporter gene expression in response to CCCTC-binding factor (CTCF).^52^ Furthermore, it has been shown that both *SLC6A4* VNTRs can bind multiple transcription factors, including y-box binding protein (YB1), CTCF and methyl-CpG binding protein (MeCP2), inducing allele-specific expression profiles in response to cocaine.^58^ A further example is the VNTR identified in the 3ʹ UTR of the human dopamine transporter (*SLC6A3*) gene, which has been associated with attention-deficit hyperactivity disorder.^40,41^ Previously it had been shown using reporter gene assays in the SH-SY5Y cell line that the *SLC6A3* VNTR induced significant repression of luciferase expression in response to the HESR (HEY) family of transcription factors, specifically HESR1 and HESR2.^59^

More recently, it has been discovered that some VNTRs have the potential to encode for miRNAs. This phenomenon has been recently described for VNTRs within the genes *WDR7* ^42^ and *DNAJC5* which harbors the human-specific miR-941 within an intronic VNTR.^48^ This latter example displays unusual features for a recently emerged human-specific miRNA, namely high levels of expression, particularly of note with regard to this review, in the cerebellum and prefrontal cortex and the copy number demonstrates a high level of variability. Human-specific regulation by this miRNA has been shown in the brain, targets included the host gene *DNAJC5* whose protein has amongst other functions a role in neurotransmitter release,^48^ preventing neurodegeneration ^60^ and is associated with adult-onset neuronal ceroid lipofuscinosis.^61^ Furthermore, this poses the question does the copy number of the VNTR leads to distinct levels of the miRNA directly, or is the mechanism indirect, achieved by modification of the processing of the internal miRNA? The putative miRNA encoded within *WDR7* (incidentally itself a target of miR-941^48^) has been detected in cytoplasmic aggregates or speckles when experimentally over-expressed. Such RNA foci, albeit in these cases nuclear, are recognized as important features in a number of RNA gain of function disease models such as that for myotonic dystrophy ^62^ and *C9ORF72-*associated ALS.^63,64^

Epigenetic modification of a VNTR could have significant consequences for all VNTR-directed regulatory mechanisms in both the immediate response to cellular signalling and the medium- and long-term properties of the VNTR to modulate gene function. This was illustrated by the analysis undertaken by Vasiliou *et al.* regarding the response of the *SLC6A4* serotonin transporter gene to stimulation by cocaine—differential effects on transcription factor binding were seen dependant on which VNTR allele was being examined and correlated with MeCP2 binding.^58^ One clear mechanism is methylation of the VNTR itself, this requires the VNTR to contain CpG targets. Simplistically, the more CpG in the VNTR, the greater opportunity for methylation changes to alter VNTR function. This is perhaps obvious in short repeats of CG, but can also occur in expansion repeats such as the hexamer CCCCGG tandem repeat found in *C9ORF72.*^65,66^ Changes in the status of CpG methylation have long been noted to affect the stability of repetitive elements containing such sequences; furthermore, methylation status has been purported to enable DNA to adopt alternative non-B DNA structural forms such as Z-DNA.^67^ Consideration of the methylation status of the VNTR may help explain some of the conflicting reports of the association of repetitive elements with traits and disease. A prime example of this is the *MAOA* gene reviewed by Ziegler and Domschke, and this highlighted that the µVNTR lies in an area that exhibits differential methylation; furthermore, the level of methylation is postulated to contribute to the different disease profiles that have been attributed to the *MAOA* gene, and the location of *MAOA* on the X chromosome could also contribute to the gender differences seen in the various analyses.^68^ This observation was expanded by Manca *et al.,* who demonstrated in a cell line model that there was allele-specific gene expression and transcription factor binding with corresponding allele-specific epigenetic marks in the region encompassing the µVNTR; furthermore, exposure to the mood stabilizer sodium valproate resulted in an allele-specific response in transcription factor binding and epigenetic marks.^54^

The final mechanism to be highlighted is modification of genome structure and in particular formation of G-quadruplexes (G4). These structures can be generated by repetition of C-rich hexameric sequences such as CCCTCC and CCCCGG and variants thereof. The classical model for G4 structure is the trimeric repeat of CCCTCCCC found in the *MYC* gene promoter which has been associated with promoter usage for MYC expression.^69,70^ Although this example is not a VNTR, it has laid the basis for the putative action of such repeats found in VNTRs in mechanisms involved in disease progression or initiation as exemplified by the CCCCGG expansion repeat in the *C9ORF72* gene associated with ALS.^71^ Such repeats are postulated to form a genomic structure that allows for torsional strain on one side and opening of the genome on the other thus allowing for a range of distinct structural parameters to be imposed at local regions of the genome. A number of transcription factors, both double stranded DNA binding factors such as CTCF and Sp1, and single stranded nucleic acid binding factors, such as hnRNPK and YB1, could then bind to such VNTRs to determine localized genomic structure.

## VNTRs in non-long terminal repeat retrotransposons

**Figure 2.**
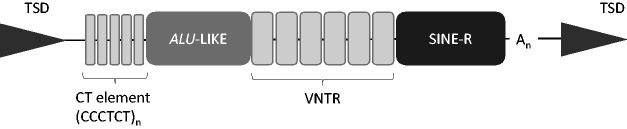
Illustration of the consensus structure of the non-long terminal repeat retrotransposon SVA element (∼0.7–4 kb). The VNTR and CT elements can both be polymorphic for the number of tandem repeats and individual single repeat elements can also be polymorphic with SNPs and/or indels, and the polyA (A_n_) may also be polymorphic in length. The element is flanked by target site duplications (TSD).

VNTRs are often considered as standalone elements in the genome but as discussed earlier they are also key domains of larger functional components such as the compound SINE-VNTR-*Alu* (SVA) element, a non-long terminal repeat retrotransposon. Although the total number of SVAs in the human reference genome is modest (approx. 2700), SVAs are hominid-specific elements with almost 50% of those characterized in the reference genome found to be human specific and have been postulated to correlate with the development of hominid lineage-specific traits.^2^ They are composite regulatory DNA domains containing multiple regulatory components (Figure 2) that affect reporter gene expression.^72^ Several of these domains are tandem repeats, specifically the CT hexamer flanking domain and a central large VNTR domain. The VNTR component of the SVA is variable in length, and it can consist of a single VNTR or two separate VNTRs (which are not necessarily both variable in number) which may share related primary sequence but are clearly distinct from one another. The VNTR may also contain more than one specific repeat motif. SVAs also have the potential to form the structural components outlined above, namely G-quadruplexes, and this ability is embedded in the CT hexamer and the central VNTR region where the CpG content can approach 60%.

The association of SVAs with disease may not only be a modifying parameter but can also be causative of the disease. This supports the main drive of our hypothesis that VNTRs are not only biomarkers of disease but can also be mechanistically involved in progression of the disease. This is demonstrated by the observation that some SVAs can be polymorphic for their presence or absence in the genome, thus for specific SVAs this means that they can be (1) associated with methylation differences at adjacent promoters as exemplified by the *LRIG2* gene,^73^ (2) associated with differential RNA expression and disease severity in Parkinson’s disease (Pfaff *et al.* in preparation), and (3) causative of disease as seen in X chromosome-linked dystonia Parkinsonism.^46^

## Summary

This review highlights the emerging field analyzing the role and importance of VNTRs in regulation of genome function and regulation of the cell transcriptome. These elements have the potential to function in many different ways and have been postulated to contribute to human evolution. It is apparent, however, that each individual element should not be considered alone, rather it is their concerted action that is important, and this provides a layer of intricacy to regulation allowing an exquisite and specific response to a stimulus. In the recent past, VNTRs have also been found to be linked with many neurological conditions and disorders and their activity is expected to explain some of the missing heritability in such disorders. To date, analysis of VNTRs in association studies has often relied on labor intensive techniques such as polymerase chain reaction (PCR), which itself can be technically challenging in these domains due to their repetitive nature and in some instances, high GC content. However, with the advent and increased availability of long-read sequencing methods, these problems will in part be overcome and allow a more rapid and robust analysis thus aiding the identification of a host of genomic and functional mechanisms underlying our physiology. For example, long-read sequencing has recently enabled the identification of a short tandem repeat (GGC) expansion in the *NOTCH2NLC* locus which can have as many as 517 repeats in affected individuals with neuronal intranuclear inclusion disease-related disorders^38^ and facilitated epigenomic profiling of transposable elements.^74^
